# Characteristics and Outcome Determinants of Hospitalized Older Patients with Cognitive Dysfunction

**DOI:** 10.3390/ijerph19010584

**Published:** 2022-01-05

**Authors:** Yi-Ting Chao, Fu-Hsuan Kuo, Yu-Shan Lee, Yu-Hui Huang, Shuo-Chun Weng, Yin-Yi Chou, Chu-Sheng Lin, Shih-Yi Lin

**Affiliations:** 1 Center for Geriatrics & Gerontology, Taichung Veterans General Hospital, Taichung 407219, Taiwan; wereyvette@vghtc.gov.tw (Y.-T.C.); kfs0611@gmail.com (F.-H.K.); yslee@vghtc.gov.tw (Y.-S.L.); hyhui@vghtc.gov.tw (Y.-H.H.); j01i02m3@yahoo.com.tw (S.-C.W.); yinyichou@gmail.com (Y.-Y.C.); billlin@vghtc.gov.tw (C.-S.L.); 2 Division of Neurology, Taichung Veterans General Hospital, Taichung 407219, Taiwan; 3 Department of Nursing, Taichung Veterans General Hospital, Taichung 407219, Taiwan; 4 Division of Nephrology, Department of Internal Medicine, Taichung Veterans General Hospital, Taichung 407219, Taiwan; 5 Institute of Clinical Medicine, College of Medicine, National Yang Ming Chiao Tung University, Taipei 112201, Taiwan; 6 Division of Allergy, Immunology and Rheumatology, Department of Internal Medicine, Taichung Veterans General Hospital, Taichung 407219, Taiwan; 7 Department of Family Medicine, Taichung Veterans General Hospital, Taichung 407219, Taiwan; 8 Division of Endocrinology and Metabolism, Department of Internal Medicine, Taichung Veterans General Hospital, Taichung 407219, Taiwan

**Keywords:** dementia, cognitive impairment, geriatric assessment, activity of daily living, length of stay, readmission

## Abstract

Cognitive dysfunction commonly occurs among older patients during admission and is associated with adverse prognosis. This study evaluated clinical characteristics and outcome determinants in hospitalized older patients with cognitive disorders. The main outcomes were length of stay, readmission within 30 days, Barthel index (BI) score at discharge, BI score change (discharge BI score minus BI score), and proportion of positive BI score change to indicate change of activities of daily living (ADL) change during hospitalization. A total of 642 inpatients with a mean age of 79.47 years (76–103 years) were categorized into three groups according to the medical history of dementia, and Mini-Mental State Examination (MMSE) scores at admission. Among them, 74 had dementia diagnosis (DD), 310 had cognitive impairment (CI), and 258 had normal MMSE scores. Patients with DD and CI generally had a higher risk of many geriatric syndromes, such as multimorbidities, polypharmacy, delirium, incontinence, visual and auditory impairment, fall history, physical frailty. They had less BI score, BI score change, and proportion of positive BI score change ADL at discharge. (DD 70.0%, CI 79.0%), suggesting less ADL change during hospitalization compared with those with normal MMSE scores (92.9%; *p* < 0.001). Using multiple regression analysis, we found that among patients with DD and CI, age (*p* = 0.008) and walking speed (*p* = 0.023) were predictors of discharge BI score. In addition, age (*p* = 0.047) and education level were associated with dichotomized BI score change (positive vs. non-positive) during hospitalization. Furthermore, the number and severity of comorbidities predicted LOS (*p* < 0.001) and readmission (*p* = 0.001) in patients with cognitive disorders. It is suggested that appropriate strategies are required to improve clinical outcomes in these patients.

## 1. Introduction

The current number of people with dementia is estimated to be 50 million globally; this number is expected to increase to 139 million by 2050 [1,2]. This increase will place a heavy burden on health-care systems, and dementia has considerable physical, psychological, and financial effects on both families and society as a whole. Previous studies have estimated the prevalence of dementia to be between 12.9% and 63.0% in the hospital [3]. People with dementia frequently experience several adverse clinical outcomes, such as longer length of stay (LOS) and higher risk of unscheduled readmission. Furthermore, the functional decline and rates of institutionalization after discharge are higher in people with dementia [4,5]. Some studies have proposed that hospitalized patients with dementia may have more comorbidities and several other disorders associated with older adults, which contribute to worse clinical outcomes after discharge [3,6].

Given the high incidence of cognitive disorders in hospitalized older patients, research exploring the factors associated with clinical outcomes is vital in guiding clinical care and preparation for discharge. However, in Taiwan, few studies have investigated outcome predictors in older patients with dementia or cognitive impairment (CI). Therefore, the aim of this study was to determine the prevalence of associated factors, namely comorbidities, functional status, and nutritional status, in hospitalized older patients with dementia or cognitive disorders and to examine the effects of these factors on change of activities of daily living (ADL) during hospitalization, LOS, and readmission after discharge.

## 2. Materials and Methods

### 2.1. Design and Participants

This study recruited patients admitted between 4 March 2016 and 20 September 2018 to the geriatric ward of Taichung Veterans General Hospital, a tertiary care academic medical center in Taiwan. Patients over 65 years old admitted due to acute illness, especially with physical function decline, multimorbidity, polypharmacy, fall, delirium, and cognitive disorders, etc. [7,8], were enrolled in our study. Some patients had previous diagnosis of dementia that was made by a neurologist or psychiatrist doctor according to the criteria of National Institute on Aging—Alzheimer’s Association (NIA—AA), or the Diagnostic and Statistical Manual of Mental Disorders (DSM)–5 criteria for major neurocognitive disorder. Patients were excluded if they were completely physically dependent before admission, had terminal illness (i.e., with an expected survival time of less than 6 months), or were limited in their ability to receive comprehensive geriatric assessment. Patients who could not complete the Mini-Mental State Examination (MMSE) were excluded, unless they had been previously diagnosed with any kind of dementia. (Such patients would be categorized into the dementia diagnosis). Finally, a total of 642 patients were included. Because all data were based on patients registered in a health system’s geriatric assessment and care database of Taichung Veterans General Hospital, and analyzed anonymously in a retrospective manner, a verbal or written consent was not required from the enrolled subjects according to the regulations from the ethics committee of the hospital. The study was approved by the ethical review committee conducted by the Institutional Review Board of Taichung Veterans General Hospital (CE18141B).

### 2.2. Assessment

Patients’ general demographic data were obtained from patients’ medical records, and included age, sex, body mass index (BMI), lifestyle habits, education level, marital status, and socioeconomic status. We further assessed patients’ medical histories and comorbidities and recorded any diagnosed diseases, medications, and age-adjusted Charlson comorbidity index (ACCI) scores [9]. At both admission and discharge, all participants were evaluated by a comprehensive geriatric assessment (CGA), as previously described. [9] Cognitive status was measured using MMSE, with scored ranging from 0 to 30, that was conducted by well-trained nurses. The normal and abnormal cognitive function cut-off points, as defined according to Taiwanese–Mini-Mental State Examination (T–MMSE), was adjusted based on age and educational level. Abnormal T–MMSE results were defined as a score < 24 in literate older adults and <14 in illiterate older adults [10]. Mood was screened using the 5-item Geriatric Depression Scale (GDS-5). Delirium is detected as the patient met the criteria of Confusion Assessment Method for the ICU (CAM-ICU). Polypharmacy is defined as more than 5 drugs used daily. Nutritional status was assessed based on Mini-Nutritional Assessment (MNA) scoring. Frailty phenotype was assessed by handgrip strength (HGS)using a handheld dynamometer (Smedley’s Dynamometer, TTM, Tokyo, Japan), and gait speed (m/s) on a 6 m course [11]. Hearing impairment is screened by the whisper test. Visual acuity is tested via Snellen chart. Physical function was assessed by Barthel Index (BI) for Activities of Daily Living (ADL), and Lawton–Brody scale for Instrumental Activities of Daily Living (IADL).

### 2.3. Outcomes

LOS and unscheduled readmission within 30 days after discharge were recorded and analyzed. Furthermore, BI scores at admission and discharge were measured. BI score at discharge, BI change (discharge BI score minus admission BI score), and proportion of positive BI score change (discharge BI score minus admission BI score > 0) were used to indicate ADL change during hospitalization.

### 2.4. Statistical Analyses

Continuous variables are expressed as median and interquartile range (IQR, 25–75%). Categorical data are expressed as numbers and percentages. Because some continuous data had a skewed distribution, comparisons were made using the Kruskal–Wallis test for continuous variables and Chi square test or Fisher’s exact test for categorical variables, respectively. To examine factors associated with length of stay and discharge BI score, it was assessed through linear regression. Factors associated with readmission and dichotomized BI score change (positive vs. non-positive) were determined by logistic regression analysis. In the simple regression analysis, the independent variables included age, sex, marital (married or non-married, including single, divorced, separated, and widowed), education level, body mass index (BMI), comorbidities, MNA, BI, MMSE scores, handgrip strength, 6-meter walking test, hearing impairment, visual impairment, falling accidents, incontinence, polypharmacy, delirium, depression, and EQ-5D-3L index. In the multiple regression, variables with *p* values of less than 0.05 from the simple regression were all included as confounders for adjustment to assess relationship between outcomes and covariates. To avoid multicollinearity, independent variables in the multiple regression models were checked by variance inflation factor (VIF) with a cutoff point < 10. The VIF values of the variables were all less than 3.0, and thus, our multiple regression models do not suffer from multicollinearity. Statistical analyses were performed using SPSS version 22.0 (SPSS, Chicago, IL, USA). Statistical significance was set at *p* < 0.05.

## 3. Results

Among the included patients, 74 had been previously diagnosed with dementia by a neurologist or psychiatrist (group with dementia diagnosis [DD]), 310 had abnormal cognitive function detected through MMSE screening (group with CI), and 258 had normal MMSE scores (group with normal cognition [N]). The demographic distribution and characteristics of each group are listed in Table 1. Compared with the N group, patients in the DD and CI groups were older, more likely to be women, had lower educational levels, had a higher prevalence of hearing impairment, and had lower BMI. In addition, patients in the DD and CI groups had higher Charlson comorbidity index scores, lower HGS (kg), slower walking speed, more falling events within 1 month, more prevalent incontinence, polypharmacy, delirium during hospitalization, and poorer nutritional status. Furthermore, compared with the N group, patients in the DD and CI groups had a higher prevalence of cerebrovascular disease, diabetes with complications, congestive heart failure, chronic obstructive pulmonary disease, and chronic kidney disease but a lower prevalence of cancer.

Regarding clinical outcomes, the LOS was slightly longer in the DD and CI groups than in the N group. Patients with DD or CI had significantly lower BI scores, BI score change at discharge, and proportion of positive BI score change during hospitalization (DD: 70.0%, CI: 79.0%, N: 92.9%, *p* < 0.001). However, no difference was found among the 3 groups regarding readmission within 30 days (DD: 5.4%, CI: 12.6%, N: 11.2%).

As presented in Table 2, for BI score at discharge, the N group as well as DD and CI group shared some common predictors, such as age, hearing impairment, MMSE scores, walking speed, MNA, EQ-5D-3L, and GDS, in the simple regression model. In the multiple regression analysis, age and gait problems were negatively associated with BIscores at discharge in the DD and CI groups. In the N group, aging was associated with poorer BI scores at discharge. Higher MMSE scores, however, were linked to higher BI scores.

As presented in Table 3, the simple regression model showed no clear determinants were observed in the N group regarding dichotomized BI score change (positive vs. non-positive) during hospitalization, and therefore, further multiple regression analysis was not examined. In the DD and CI groups, age, education level, MMSE, IADL, MNA, were associated factors with positive BI score change. In the multiple regression model, older age and higher educational levels remain to display significance. For LOS, in the patients with DD or CI, the number and severity of comorbidities predicted longer stay in the hospital (Table 4). While, in the normal group, BMI, walking speed, and numbers of comorbidities were predictors of LOS markers in the multiple regression model. Regarding unscheduled readmission within 30 days, no obvious significant predictors were identified for patients with N in the simple regression model (Table 5), whereas CCI scores and possible impaired visual function were correlated with readmission in the DD and CI groups, as shown in the simple and multiple regression model. Some variables that were not significantly associated with outcome (i.e., *p* > 0.05), such as discharge destination, were not shown in the tables.

## 4. Discussion

The findings of our retrospective study showed that the prevalence of dementia and CI in hospitalized older patients was high, and patients with dementia diagnosis and CI have more geriatric syndromes, such as frailty, gait disturbance, falling accidents, incontinence, polypharmacy, delirium, and malnutrition. Besides, patients with DD or CI had slightly longer LOS than that in the N group, lower BI scores, BI score change, and proportion of positive BI score change at discharge, indicating less ADL change or improvement during hospitalization. Furthermore, in patients with DD or CI, the number and severity of comorbidities were associated with LOS and readmission. Overall, older hospitalized patients with cognitive disorders had worse clinical outcomes compared with those without cognitive disorders [12,13,14].

In this study, patients with DD or CI exhibited further comorbidities, such as stroke, diabetes, and heart failure. Several studies have proposed that these medical disorders share a common cardiovascular risk factor for dementia and can subsequently contribute to neurodegeneration [15,16,17]. Additionally, hemodynamic instability, activated neurohormones, or oxidative stress may play a role in the development of CI [18]. In addition, patients with DD and CI in our study had a higher prevalence of chronic kidney disease, which has also been reported in previous studies [19,20]. However, the exact mechanism linking renal dysfunction to CI and dementia remains unclear; it may be related to inflammation, oxidative stress, or small vessel disease. Regarding chronic obstructive pulmonary disease, chronic hypoxemia, comorbid cardiovascular disease, and smoking were probable contributors to cognitive impairment [21,22]. Because of the frequent presence of multimorbidities in patients with dementia and cognitive disorders, we expected the numbers of medications for these patients to be higher. In our study, the frequency of polypharmacy was 76.6% in patients with DD and CI.

As in previous reports, we found that hearing impairment was associated with CI and DD. Vascular dysfunction, confounding cardiovascular comorbidities, impaired verbal communication, and temporal volume loss in the hippocampus and entorhinal cortex may play roles in the association between hearing impairment and cognitive decline [15,23,24]. In our patients, an inverse relationship was observed between cancer and DD and CI, which is in line with previous reports [25,26]. Studies have proposed that survival bias, underdiagnosis of cancer, and uninvestigated biological signal pathways may explain this association. Depression, depressive mood, or dysthymia is also frequently associated with dementia [27,28]. However, in our patients, the prevalence of depression was not high in the DD and CI groups. This is likely because the diagnosis of depressive symptoms is generally difficult in these types of patients due to their advanced stages of dementia or cognitive dysfunction. This study also found higher delirium rates in patients with cognitive disorders (DD and CI). In the literature, delirium has been reported to be more prevalent in acutely ill inpatients, especially those who were previously cognitively impaired [29,30], which consequently leads to long-term cognitive decline [31,32].

The proportion of malnourished patients with DD and CI was higher than that of patients with N, which is compatible with the findings of previous studies [33]. Several studies have reported that nutritional problems, such as appetite changes, weight loss, and sarcopenia, can develop early in patients with mild CI and early-stage Alzheimer’s disease [34,35]. Due to the inherent cognitive degeneration and gradual physical decline in patients with dementia, malnutrition may cause them to become more dependent on care from others. Furthermore, the prevalence of urinary incontinence was 42.4% in our patients with DD and CI. In previous studies, the prevalence of urinary incontinence varied considerably, from 11% to 90%, in individuals with DD [36,37]. Causes of urinary incontinence among older adults with DD include detrusor over activity (due to a lack of central inhibition), a lack of caregivers, impaired mobility, and psychological barriers associated with the unwillingness to use the toilet [36]. Compared with older people who have normal MMSE scores, older patients with DD have a more than two-fold higher rate of falls. Studies have proposed that the high rate of falls may be due to gait disorders, as demonstrated by the decline in both memory and gait speed in our cohort; frailty; and use of psychotropic drugs [38]. Therefore, health-care staff should remind patients or patients’ relatives to bring ambulatory aids when older patients are hospitalized.

Our study found that patients with DD and CI had less ADL change during hospitalization. A previous meta-analysis found that several functional measurements, including ADL, IADL, and mobility, were associated with CI or DD in hospitalized adults aged ≥65 year [39,40]. The mechanisms of these associations are likely multifactorial. First, the associated acute illnesses in patients with CI may cause functional loss through direct effects on the musculoskeletal system [39]. In addition, a pre-existing neurological impairment, such as visual or auditory dysfunction, multicomorbidities, and polypharmacy, may reduce the ability to recover from an initial illness-related functional loss. Furthermore, CI may be a marker of underlying frailty and general vulnerability. CI is also associated with decreased participation in rehabilitation, poorer functional outcomes, increased LOS, and decreased rates of discharge to home [41,42]. Notably, this study found that the educational level was negatively associated with ADL change during hospitalization, which supports the findings of prior studies that the education level has an inverse relationship with remaining independent [43]. A possible explanation is that those with higher education levels are generally wealthy and may lead a more leisurely life; they may, therefore, be more likely to be physically inactive and less likely to engage in strengthening activities, such as physical labor [43]. Overall, recognition of CI may facilitate evaluation of rehabilitation potential, rehabilitation interventions, and goal-setting, as well as planning future care.

Higher mortality, readmission risk, and LOS have been widely reported in hospitalized patients with DD [39,44]. However, this study found that readmission and LOS were similar in patients with and without cognitive disorders, which is supported by the findings of a previous study [45] that multimorbidity rather than CI predicted readmission and long LOS in patients with cognitive disorders. This result reinforced the hypothesis that the effects of multimorbidity on clinical outcomes result from the synergistic interplay of chronic disorders, lifestyle factors, aging, and consequent mental and physical impairment rather than the sum of their individual effects. Therefore, ward-based patient care might provide more efficient support for older inpatients with multimorbidities. Furthermore, interdisciplinary team care should be implemented to ensure more favorable outcomes, including short LOS, prevention of harm associated with hospitalization, and reduction in healthcare costs [46].

Given the clinical impact of cognitive disorders, we urge clinicians to be vigilant in providing timely and proper management for these patients in daily practice. The results of this study may facilitate early decision-making and to ensure advance care planning for patients, clinicians, and carers. However, an urgent need exists for further research that will improve the unfavorable prognoses in patients with CI and DD.

This study has several limitations. First, the group of patients with DD or CI is heterogeneous and contains a wide variety of patients with diverse backgrounds, underlying disorders, and comorbidities. Some influence or interaction inherent to outer environments may have occurred that affected our results. Second, we used only MMSE to screen patients for CI; more comprehensive or precise examinations or more specific imaging, laboratory testing, and genetic testing may be necessary to diagnose dementia in patients with CI. Besides, the data of disease types and severity for patients with dementia were lack. It has been reported that patients with Lewy body dementia have a shorter time between first diagnosis and first admission, a higher admission rate than AD patients, and a longer length of stay in the hospital [47], although the other study showed that frequency of hospitalization was not different among Alzheimer disease, vascular dementia, or Parkinsonism-related dementia [48]. Third, in this study, we only measured once the ADL score, at admission and at discharge. Whether there was test–retest (intra-rater) reliability and consistency on repeated testing of the same patient was not known. It has been reported that ADL measurement by the Barthel index can be generally recommended in older people, and there was a high percentage agreement for the total BI score. However, test–retest reliability remained less certain, especially in older people with multiple diagnoses [49]. Fourth, patient data were collected only from a single medical center; therefore, selection bias may not be fully eliminated. Finally, this was a retrospective observational study, and causality could not be fully identified. Further research remains necessary in order to establish a more definite conclusion.

## 5. Conclusions

In this study, we found that hospitalized older patients with cognitive disorders were at higher risk of several comorbidities and geriatric syndromes. In addition, patients with cognitive disorders demonstrated less change of ADL during hospitalization even after treatment of their medical diseases. Furthermore, the number and severity of comorbidities predicted LOS and readmission. Early identification and tailored management of risk factors leading to unfavorable outcomes are required for patients with abnormal cognitive functional status.

## Figures and Tables

**Table 1:** Characteristics and outcomes of the patients.

		Dementia Diagnosis (*n* = 74)	Cognitive Impairment (*n* = 310)	Normal (*n* = 258)	*p* Value
**Demography**					
	Age, years	86 (79–90)	81 (75–87)	75 (69–85)	<0.001
	BMI (kg/m^2^)	23.5 (20.1–25.7)	23.3 (20.5–26.7)	23.9 (21.6–27.4)	0.019
	Sex				0.035
	Male	46 (62.2)	160 (51.6)	159 (61.6)	
	Female	28 (37.8)	150 (48.4)	99 (38.4)	
	Educational level				<0.001
	Illiterate	22 (29.7)	102 (32.9)	16 (6.2)	
	Literate/primary school	24 (32.4)	130 (41.9)	92 (35.7)	
	Junior/Senior high school	18 (24.3)	57 (18.4)	99 (38.4)	
	University	10 (13.5)	21 (6.8)	51 (19.8)	
**Comprehensive Geriatric Assessment**					
	Mini-Mental Status Examination	14 (10–20)	18 (14–21)	28 (26–29)	<0.001
	Charlson comorbidity index	4 (2–5)	3 (2–4)	2 (1–4)	<0.001
	Number of comorbidities	3 (2–4)	2 (1–3)	1 (1–2)	<0.001
	Handgrip strength (kilogram)				
	Male	14.6 (11.3–20.8)	18.0 (12.5–23.4)	25.5 (19.8–30.5)	<0.001
	Female	9.0 (6.8–11.4)	12.3 (8.6–15.9)	15.1 (11.8–18.1)	<0.001
	6-meter walking test (seconds)	14.8 (10.7–23.9)	14.2 (9.9–22.4)	10.6 (7.8–15.6)	<0.001
	Hearing impairment	31 (41.9)	107 (34.5)	63 (24.4)	0.002
	Visual impairment	33 (44.6)	160 (51.6)	149 (57.8)	0.078
	Fall within 1 month	36 (48.7)	147 (47.4)	49 (19.0)	<0.001
	Fall within 1 year	42 (56.8)	176 (56.8)	62 (24.0)	<0.001
	Urine incontinence	38 (51.4)	125 (40.3)	77 (29.8)	0.001
	Stool incontinence	7 (9.5)	39 (12.6)	14 (5.4)	0.014
	Polypharmacy	59 (79.7)	235 (75.8)	176 (74.0)	0.002
	Delirium	4 (5.5)	13 (6.2)	0	0.001
	Depression	22 (31.0)	125 (40.9)	85 (33.0)	0.055
	Mini Nutritional Assessment				<0.001
	<17	29 (39.2)	73 (23.6)	20 (7.8)	
	17~23.5	33 (44.6)	166 (53.6)	101 (39.2)	
	>24	12 (16.2)	71 (22.9)	137 (53.1)	
**Comorbidities**					
	Hypertension	52 (70.3)	227 (72.2)	173 (67.1)	0.127
	Diabetes mellitus	27 (36.5)	119 (38.4)	87 (33.7)	0.304
	Cerebrovascular disease	31 (41.9)	82 (26.5)	26 (10.1)	<0.001
	Diabetes with end organ damage	22 (29.7)	90 (29.0)	43 (16.7)	<0.001
	Congestive heart failure	19 (25.7)	98 (31.6)	34 (13.2)	<0.001
	Chronic obstructive pulmonary disease	22 (29.73)	106 (34.2)	60 (23.3)	0.006
	Pneumonia	20 (27.0)	71 (22.9)	55 (21.3)	0.481
	Chronic kidney disease	7 (9.5)	55 (17.7)	28 (10.9)	0.058
	Cirrhosis	0 (0)	7 (2.3)	3 (1.2)	0.508
	Gastrointestinal disease	18 (24.3)	59 (19.0)	66 (25.6)	0.099
	Cancer	3 (4.1)	26 (8.4)	49 (19.0)	<0.001
**Outcome**					
	Barthel index (BI) score at discharge *	35 (20–70)	55 (30–75)	80 (65–90)	<0.001
	BI score change * (discharge BI minus admission BI)	10 (0–15)	10 (5–20)	15 (5–20)	0.002
	Proportion of BI score change > 0 vs. ≤0 *				
	BI score change ≤ 0	18 (30.0)	49 (21.0)	14 (7.1)	<0.001
	BI score change > 0	42 (70.0)	184 (79.0)	184 (92.9)	<0.001
	Length of stay	10 (7–15)	10 (7–15)	9 (7–14)	0.116
	Readmission within 30 days	4 (5.4)	39 (12.6)	29 (11.2)	0.213

Data are expressed as median (IQR) for continuous variables and N (%) for categorical variables. * DD (*n* = 60), CI (*n* = 233), N (*n* = 198).

**Table 2:** Predictors of discharge BI scores.

	Simple Regression						Multiple Regression					
	Normal			Dementia + Cognitive Impairment			Normal			Dementia + Cognitive Impairment		
	B	Beta (β)	*p*	B	Beta (β)	*p*	B	Beta (β)	*p*	B	Beta (β)	*p*
Age	0.00	−0.15	0.030	−0.01	−0.24	<0.001	0.00	−0.32	0.001	−0.004	−0.254	0.008
Gender												
Male	Ref			Ref								
Female	−0.03	−0.09	0.204	0.12	0.13	0.022						
BMI (kg/m^2^)	0.00	0.06	0.388	0.02	0.15	0.017						
Main caregiver												
Self	Ref			Ref								
Spouse	−0.04	−0.09	0.094	−0.32	−0.28	<0.001						
Descendants	−0.10	−0.20	<0.001	−0.20	−0.22	<0.001						
Polypharmacy	−0.02	−0.07	0.348	−0.17	−0.16	0.005						
Hearing impairment	−0.05	−0.19	0.007	−0.11	−0.13	0.033						
Visual impairment	−0.04	−0.13	0.072	−0.14	−0.15	0.009						
Mini-mental state examination	0.02	0.22	0.002	0.04	0.44	<0.001	0.01	0.40	<0.001			
Charlson comorbidity index	0.00	−0.03	0.681	−0.01	−0.07	0.252						
6- meter walking test	0.00	−0.24	0.005	0.00	−0.36	<0.001				−0.002	−0.230	0.023
Mini Nutrition Assessment	0.02	0.39	<0.001	0.05	0.50	<0.001						
EQ-5D-3L index	0.25	0.55	<0.001	0.05	0.50	<0.001						
Handgrip strength (kg)-Female	0.00	0.31	<0.001	0.02	0.29	<0.001						
Handgrip strength (kg)-Male	0.00	0.10	0.411	0.02	0.31	0.001						
5-item Geriatric Depression Scale ≥ 2	−0.07	−0.23	0.001	−0.12	−0.13	0.034						

Ref., reference; B, unstandardized coefficients; Beta, standardized coefficients; BMI, body mass index; EQ-5D-3L index, 3-level version of European Quality of life-5 dimensions index.

**Table 3:** Associated factors of dichotomized BI score change (positive vs. non-positive).

	Normal			Dementia + Cognitive Impairment					
	Simple Regression Model			Simple Regression Model			Multiple Regression Model		
	OR	95% CI	*p*	OR	95% CI	*p*	OR	95% CI	*p*
Age	0.98	0.92–1.04	0.514	0.94	0.90–0.97	0.001	0.96	0.92–1.00	0.047
Gender									
Male	Ref			Ref					
Female	0.73	0.24–2.19	0.572	1.54	0.88–2.69	0.132			
BMI (kg/m^2^)	0.89	0.79–1.00	0.052	1.05	0.97–1.12	0.235			
Education									
Illiterate	Ref			Ref					
Literate/primary school	0.00	0..00	0.999	0.44	0.21–0.91	0.026	0.36	0.16–0.78	0.010
Junior/senior high school	0.00	0.00	0.999	0.43	0.18–0.99	0.048	0.42		
University	0.00	0.00	0.999	0.27	0.10–0.75	0.012	0.28	0.09–0.88	0.029
Mini-mental state examination	1.26	0.91–1.73	0.160	1.10	1.04–1.16	<0.001	1.07		
Lawton scale (IADL)	0.92	0.71–1.18	0.497	1.47	1.20–1.81	<0.001	1.20		
Mini Nutritional Assessment	0.99	0.84–1.17	0.934	1.09	1.03–1.16	0.005	1.01		

OR, odds ration; CI, confidence interval; Ref., reference; BMI, body mass index; IADL, instrumental activities of daily living.

**Table 4:** Predictors of Length of Stay.

	Normal						Dementia + Cognitive Impairment					
	Simple Regression			Multiple Regression			Simple Regression			Multiple Regression		
	B	Beta (β)	*p*	B	Beta (β)	*p*	B	Beta (β)	*P*	B	Beta (β)	*p*
Age	−0.069	−0.076	0.252				−0.193	−0.130	0.011			
Gender												
Male	Ref						Ref					
Female	0.022	0.001	0.984				−1.981	−0.082	0.106			
BMI (kg/m^2^)	−0.284	−0.149	0.030	−0.389	−0.221	0.004	0.008	0.003	0.954			
Mini-mental state examination	−0.334	−0.071	0.284				0.056	0.025	0.632			
Charlson comorbidity index	1.150	0.314	<0.001	1.112	0.307	<0.001	1.559	0.307	<0.001			
Number of comorbidities	1.879	0.294	<0.001				2.521	0.317	<0.001	2.058	0.304	<0.001
6-meter walking test	0.157	0.204	0.013	0.137	0.181	0.017	0.004	0.006	0.945			
Mini Nutritional Assessment	−0.492	−0.224	0.001				−0.251	−0.095	0.063			
EQ-5D-3L index	−5.095	−0.210	0.001				−0.106	−0.004	0.942			

Ref., reference; B, unstandardized coefficients; Beta, standardized coefficients; BMI, body mass index; EQ-5D-3L index, 3-level version of European Quality of life-5 dimensions index.

**Table 5:** Associated Factors of Readmission within 30 days.

	Normal			Dementia + Cognitive Impairment					
	Simple Regression			Simple Regression			Multiple Regression		
	OR	95% CI	*p* Value	OR	95% CI	*p* Value	OR	95% CI	*p* Value
Age	1.05	1.00–1.09	0.056	1.00	0.96–1.04	0.858			
Gender									
Male	Ref			Ref					
Female	0.91	0.39–2.14	0.827	0.52	0.27–1.20	0.057			
BMI (kg/m^2^)	1.02	0.93–1.13	0.676	0.98	0.90–1.06	0.596			
Hearing impairment	1.21	0.48–3.05	0.690	0.95	0.49–1.84	0.868			
Visual impairment	1.44	0.61–3.39	0.402	1.96	1.01–3.80	0.047	2.05	0.99–4.22	0.052
Mini-mental state examination	1.19	0.93–1.53	0.164	1.01	0.95–1.07	0.770			
Charlson comorbidity index	1.16	0.98–1.39	0.091	1.23	1.09–1.39	0.001	1.25	1.10–1.42	0.001

OR, odds ratio; CI, confidence interval; Ref., reference; BMI, body mass index.

## Data Availability

Not applicable.
